# Dynamics of Spring Regrowth and Comparative Production Performance of 50 Autumn-Sown Alfalfa Cultivars in the Coastal Saline Soil of North China

**DOI:** 10.3390/life11121436

**Published:** 2021-12-20

**Authors:** Shichao Wang, Dong Fang, Asif Ameen, Xiaolin Li, Kai Guo, Xiaojing Liu, Lipu Han

**Affiliations:** 1 Key Laboratory of Agricultural Water Resources, Hebei Key Laboratory of Soil Ecology, CAS Engineering Laboratory for Efficient Utilization of Saline Resources, Center for Agricultural Resources Research, Institute of Genetic and Developmental Biology, Chinese Academy of Sciences, Shijiazhuang 050022, China; scwang@sjziam.ac.cn (S.W.); fangdong19@mails.ucas.ac.cn (D.F.); xlli@ms.sjziam.ac.cn (X.L.); guokai@sjziam.ac.cn (K.G.); xjliu@sjziam.ac.cn (X.L.); 2 University of Chinese Academy of Sciences, Beijing 100049, China; 3 Rice Programme, Pakistan Agricultural Research Council, Kala Shah Kaku, Lahore 39020, Pakistan; asifameen2007@gmail.com

**Keywords:** soil salinity, autumn sown, alfalfa growth, harvest frequency, subordinate function value

## Abstract

Alfalfa (*Medicago sativa* L.) production is affected by many factors, including management practices, soil conditions, and the environmental elements of the target area. Varietal differences, in terms of agronomic performance and forage yield, among 50 alfalfa cultivars under six harvest systems following regrowth were evaluated during the growing season of 2019–2020 under non-irrigated rainfed conditions in a coastal saline-alkali soil region of North China. Days to harvesting, plant height, canopy area, growth rate, and forage yield were assessed to rank the cultivars. Furthermore, the key factor influencing the regrowth of the second year after over-wintering was identified based on the growth status before over-wintering by using the Boston Matrix method. Results showed significant (*p* < 0.05) differences among cultivars and harvests regarding plant height, canopy area, and forage yield. Alfalfa forage yield ranged between 24.2 t ha^−1^ yr^−1^ and 32.7 t ha^−1^ yr^−1^. The highest forage yield was obtained in cultivar Guochan No.1, and was lowest in cultivar Magnum 601. Forage yield reached the greatest values for the first harvest, and then decreased gradually and changed stably. The forage yield of the third, fourth, fifth, and sixth harvest ranged from 3.4 t ha^−1^ to 4.3 t ha^−1^ (averaged across 50 cultivars), which represented 10.8% to 15.2% of the annual total forage production. We also observed that forage yield correlated strongly, but negatively, with the growth rate. According to subordinate function value analysis, Womu No.1, WL440HQ, Weston, Surprise, and WL354HQ proved optimum cultivars for general cultivation in this coastal area. In future, development of alfalfa cultivars with improved regrowth and tolerance to heavy saline-alkali soil and early spring drought would be necessary to increase forage yield under rainfed conditions in coastal saline-alkali areas of North China.

## 1. Introduction

In recent years, livestock production has been increased rapidly, particularly in North China [1], which is highly dependent on adequate forage resources. According to the China Rural Statistical Yearbook [2], total livestock population in China is estimated to be 544.2 million pigs, 45.3 million cattle, 316.9 million sheep, and 14,640.6 million poultry. Grassland accounts for 40% of Chinese land, which has seen a loss of 22.7% from 1982 to 2010 [3]. The shortage of forage seriously restricts livestock husbandry. To ensure sufficient forage supplies, high quality forage of the high-yielding and salt and drought-tolerant cultivars is vigorously needed to be developed [4,5].

Saline-alkali land is a potential land resource in China, occupying an area of 3.6 × 10^7^ ha^−1^, which covers about 3.8% of the global area of saline-alkali land and 4.9% of the total national land [6]. A large area of saline-alkali soil is located in the coastal regions of Northern China [7,8]. However, high salt concentrations, a shallow saline groundwater table, freshwater shortages, and high evaporation are the major limitations for normal and optimum crop production in these coastal regions [7,9,10]. An early spring with poor rainfall would not leach salts out of the root zone, which can severely restrict crop productivity [4,11]. Therefore, cultivation of salt- and drought-tolerant forage plants is particularly important in this region in order to better utilise these land resources to meet the demands of the livestock population.

Alfalfa (*Medicago sativa* L.) exhibits a high adaptability to a range of environments, resulting in widespread growth across the different continents of the world [12]. At the same time, it plays an important role in forage production in arid and semiarid areas because of its high forage yield and nutritive value, as well as tolerance to drought and salt [13,14], and it is known as the “Queen of Forages”. There are two reasons for this; alfalfa with deep roots can be widely grown to meet the increasing demand of forage for the increasing livestock population and to prevent soil salinization in the water-limited and saline regions across the world. Specifically, the tolerant alfalfa cultivars had a relatively high level of root length, root surface area, and root volume [15]. Moreover, alfalfa can also tolerate frequent harvestings [16,17]. Thus, the production area of alfalfa is being enhanced every year globally [18,19]. So far, more than 4.0 × 10^6^ t ha^−1^ yr^−1^ of alfalfa forage has been grown and harvested from the northern and north western regions of China [17]. However, freshwater shortages and soil salinization are the major obstructions to alfalfa growth in these regions [20].

Alfalfa planting and cultivar selection should be primarily recommended, keeping in view the edaphic and environmental conditions of a certain region [11,21], with soil, rainfall, and air temperature as the most important contributors [21]. Salinization is a frequent productivity-limiting factor due to the water deficit. Moreover, alfalfa productivity is affected by its fall dormancy level that has correlation with forage yield, agronomic characteristics, and nutritive value [22]. Harvest interval or frequency also affects alfalfa growth characteristics and forage yield [23]. The harvest interval or frequency is influenced by environmental factors [24]. Generally, it is widely reported that increasing cutting intervals gives higher forage yield because of the higher proportion of stem [24]. Besides this, spring regrowth was also related to the autumn sowing period and growth status before over-wintering [25,26]. Thus, understanding about these factors affecting alfalfa growth is not only important for cultivar selection, but is also useful to improve forage yield.

Considering past studies, it was a great challenge to develop new alfalfa cultivars which had good agronomic traits for the purpose of livestock production [27]. So far, little research has been done on the agronomic performance of autumn-sown alfalfa in coastal regions and how over-wintering affects plant regrowth the following year. The variations in the agronomic performances of diverse alfalfa cultivars, with different harvest frequencies, weather conditions, and fall dormancy levels in shallow groundwater conditions of saline-alkali areas, are needed to be critically investigated further. Our hypothesis is that the agronomical performance and forage yield of alfalfa may change with cultivars, weather conditions, and fall dormancy levels and that alfalfa regrowth during the second year may be affected by growth before over-wintering when alfalfa is planted in the autumn season. Therefore, the main objectives of this study were to analyse the effects of alfalfa growth before over-wintering on its regrowth during the second year, and to evaluate the performance of 50 alfalfa cultivars regarding their agronomic traits and forage yield under rainfed conditions, in order to facilitate the selection of high-yielding cultivars that adapted to this study area.

## 2. Materials and Methods

### 2.1. Description of Experimental Site

**Figure 1:**
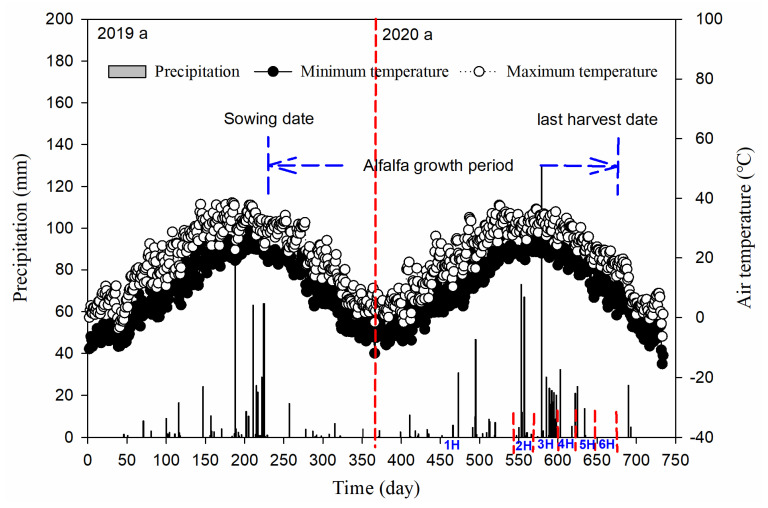
Daily precipitation and the minimum and maximum air temperature during the alfalfa growing period from 2019 to 2020 (Source: Chinese Meteorological Bureau). (1H): first harvest; (2H): second harvest; (3H): third harvest; (4H): fourth harvest; (5H): fifth harvest; (6H): sixth harvest.

The field experiment was conducted in Nandagang County (117°22′ E, 38°28′ N) of Hebei Province in Northern China between August 2019 and October 2020. The study area belongs to a typical temperate continental monsoon-type climate. The average annual precipitation was 590 mm, which occurs mostly between June and September, while mean annual evaporation was 1950 mm. The groundwater table exists at approximately 0.3–1.2 m, with a salt concentration of 6 to 10 g L^−1^. The soil in our experimental site had a silty clay loam texture. Weather data were collected from the China Meteorological Data Network, and the daily precipitation and maximum and minimum temperature during 2019 and 2020 are presented in Figure 1. In 2019, total precipitation was 556.6 mm and maximum and minimum mean air temperature was 9.5 °C and 19.7 °C, respectively. In 2020, total precipitation was 748.0 mm and maximum and minimum mean air temperature was 9.6 °C and 19.3 °C, respectively.

### 2.2. Experimental Design and Field Management

A total of 50 alfalfa cultivars were tested for agronomic performance and forage yield using randomised block design (RBD) with three replications. These 50 cultivars represented themselves as 50 different treatments, and the details regarding the dormancy level, origin, and seed source of each cultivar is presented in Table 1. Each alfalfa cultivar was planted in a separate plot with a net size of 16 m^2^ (4 m × 4 m). Sowing was done on 20 August 2019. A basal dose of 70.1 kg N ha^−1^ as urea and 80.7 kg P_2_O_5_ ha^−1^ as diammonium phosphate was applied just before the seed sowing on the day of planting. Afterwards, 22.7 kg ha^−1^ potassium dihydrogen phosphate was continuously applied after each harvest. No irrigation water was applied during the entire experimental period. Crop management was kept uniform in all the experimental plots.

In 2019, the alfalfa was cut once on 15 October before over-wintering at the stubble height of 5 cm. Aboveground plants were harvested six times in total on the dates of 25 October in 2020, and on 12 May, 4 June, 4 July, 27 July, 27 August, and 26 September in 2020. The alfalfa was harvested when alfalfa reached 20% bloom stage. At each harvest, aboveground plants were mowed at the height of 5 cm from the ground level to facilitate fast regrowth.

### 2.3. Sampling and Measurements

To monitor the plant height of alfalfa (AH, cm), five plants from five different points in each plot were randomly measured from the ground level to the top of the plants using a ruler every 5 days. Growth rate was calculated using the formula (AH_2_ − AH_1_)/(d_2_ − d_1_), where AH_2_ and AH_1_ represent the plant height measured at the day d_2_ and d_1_, respectively. Canopy projection area (CPA) affects alfalfa growth dynamics. The CPA was measured when alfalfa was growing vigorously (CPA = length × height). Three 0.7 × 1 m quadrat were taken from each experimental plot to estimate fresh biomass yield. The aboveground dry biomass of harvested samples from each plot was determined by oven-drying at 70 °C until a constant weight was reached. Forage yield of alfalfa was calculated on dry-weight basis. Total annual yield for each cultivar was determined by adding the biomass yield from all six harvests.

### 2.4. Data Analysis

#### 2.4.1. Boston Matrix Method

The difference of alfalfa growth was analysed by using two parameters, namely growth status (forage yield, plant height, canopy area, and growth rate). These two parameters were used to reflect the effects of growth status before over-wintering (when alfalfa was planted in autumn in different cultivar) on regrowth in the second year by applying the Boston Matrix method [28].

#### 2.4.2. Subordinate Function Value

Production adaptability involving multiple indices i.e., plant height, canopy area, growth rate, and forage yield, were evaluated based on their subordinate function value (SFV) [29] which is shown in formula (1) as following:

SFV = (*X* − *A_min_*)/(*X_max_* − *X_min_*) (1)

where *X* is the comprehensive value of each index of alfalfa cultivars; *X_max_* and *X_min_* are maximum and minimum values for comprehensive index, respectively.

All the data were presented as mean ± standard deviation (SD) of three replications. Differences among treatments were separated by the least significance difference (LSD) test at 0.05 significance level using the SAS (8.0) software package. Correlations and regression analysis were performed using SPSS 17.0. The figures were plotted using SigmaPlot 12.5. Boston Matrix analysis was performed using MS Excel 2020.

## 3. Results

### 3.1. Alfalfa Growth Period and Forage Yield

Differences were observed in the alfalfa growth period of all 50 cultivars (Table 2). The seedling emergence time ranged between 1 and 6 days, with the first germinations appearing in cultivars AC4, AC14, AC19, and AC27. The earliest flowering appeared in the cultivars AC3, AC12, AC13, AC14, AC21, AC22, AC31, and AC32, and the variation ranged from 1 to 5 days. The vegetative stage was the longest in cultivar AC10, while shortest vegetative stage was observed in cultivars AC20, AC21, AC22, AC31, AC32, AC34, AC39, and AC40.

**Figure 2:**
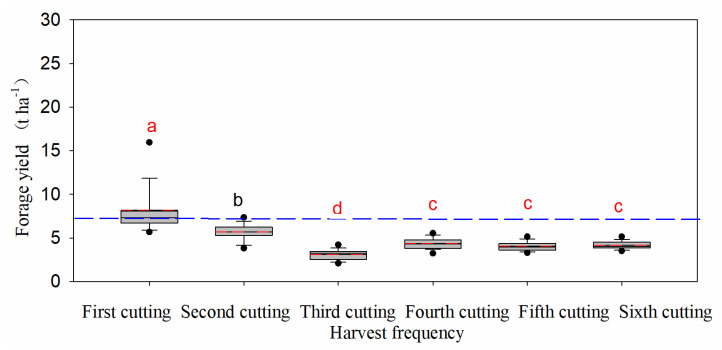
Average forage yield of alfalfa during each cutting date (*n* = 150). Different letters indicate significant difference among means at *p* < 0.05. Solid and dashed lines in the figure indicate mean and median forage yield. The lower and upper boundaries, bars, and dots in or outside the boxes indicate 25th and 75th, 5th and 95th. Numbers are shown in parentheses.

**Figure 3:**
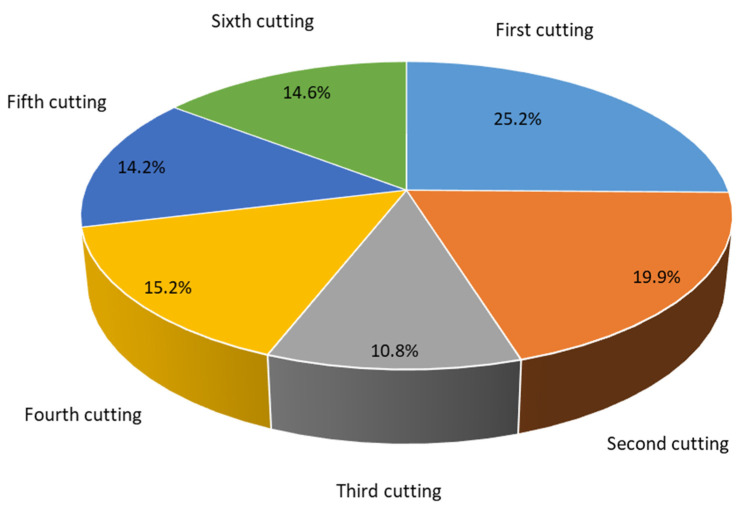
Percentage of alfalfa forage yield during each cutting date.

Overall, the forage yield of alfalfa from the first to sixth cutting decreased significantly (*p* < 0.05), and tended to adapt to a gradual decreasing trend (Figure 2 and Figure 3). The highest forage yield was recorded in the first cutting (7.2 t ha^−1^), while the lowest was recorded in the third cutting (3.1 t ha^−1^), accounting for 25.2% and 10.8%, respectively, of the total annual forage yield. Results further indicated that the effect of alfalfa cultivars on total annual forage yield was highly significant (*p* < 0.05; Table 3). Cultivars AC1, AC13, AC7, AC5, and AC21 ranked in the top five regarding the total annual forage yield, which produced forage yields of 32.7 t ha^−1^, 31.7 t ha^−1^, 31.4 t ha^−1^, 31.4 t ha^−1^, and 31.3 t ha^−1^, respectively. The correlation analysis between the total annual yield and agronomic traits showed that the total annual yield was not associated with plant height and growth rate, but it was negatively correlated with the growth rate (r = −0.300, *p* < 0.05) (Table 4). On the other hand, plant height was positively associated with both canopy area and growth rate (r = 0.926, *p* < 0.01; r = 0.312, *p* < 0.05; respectively).

**Figure 4:**
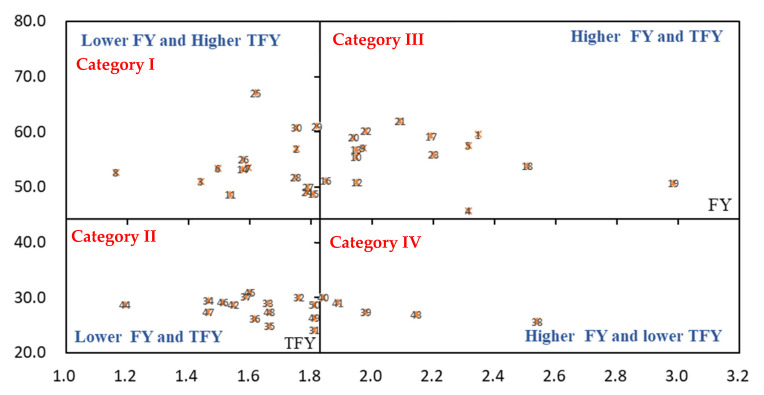
BCG matrix-based analysis of forage yield (*n* = 50). Different numbers of present alfalfa cultivars in this figure. FY: forage yield before over-wintering in 2019; TFY: total forage yield of all six harvests.

As shown in Figure 4, there were 15 cultivars in Category I, namely, WL440, Ameri graze 37 CR, and Magnum551, etc. The forage yield before over-wintering was not high, but their total forage yield in the second year was higher. There were 15 cultivars located in Category II, including Magnum601, SK4020, Phabulous, and Magnum7, etc. These cultivars were not good both in forage yield before over-wintering and in the second year. Similarly, there were 15 cultivars in Category III, namely Seniti, ladino, WL903HQ, and WL298HQ, etc. These cultivars had a higher forage yield before over-wintering and a higher total forage yield in the second year. Furthermore, there were five cultivars in Category IV, namely Magnum7, Eureka, WL656HQ, Khan, and SK3010. They had a higher forage yield before over-wintering, but low total forage yield during the second year.

### 3.2. Plant Height

**Figure 5:**
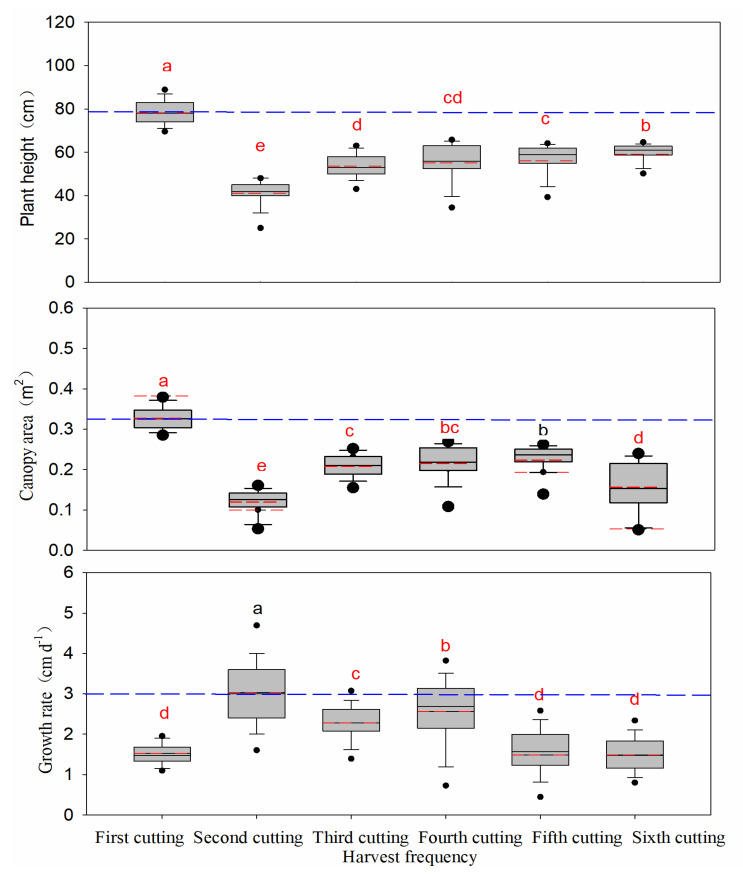
Plant height, canopy area, and growth rate of alfalfa during different cutting dates (*n* = 150). For each plant height, canopy area, and growth rate different letters indicate significant difference among means at *p* < 0.05. Solid and dashed lines in three figures indicate mean and median plant height, canopy area, and growth rate. The lower and upper boundaries, bars, and dots in or outside the boxes indicate 25th and 75th, 5th and 95th. Numbers are shown in parentheses.

There were significant differences in plant heights during different cutting dates (*p* < 0.05), and the order was as following: first cutting > sixth cutting > fifth cutting > fourth cutting > third cutting > second cutting (Figure 5). When compared with the first cutting, the mean plant height of alfalfa in the second, third, fourth, fifth, and sixth cutting decreased by 47.4%, 31.4%, 29.5%, 28.2%, and 24.4%, respectively. The overall mean plant height for all 6 cuttings ranged from 29.9 to 62.0 cm. The tallest cultivars were AC25 (62.0 cm) and AC14 (61.7 cm), whereas the shortest cultivar was AC10 with a height of 29.9 cm (*p* < 0.05) (Table 5).

**Figure 6:**
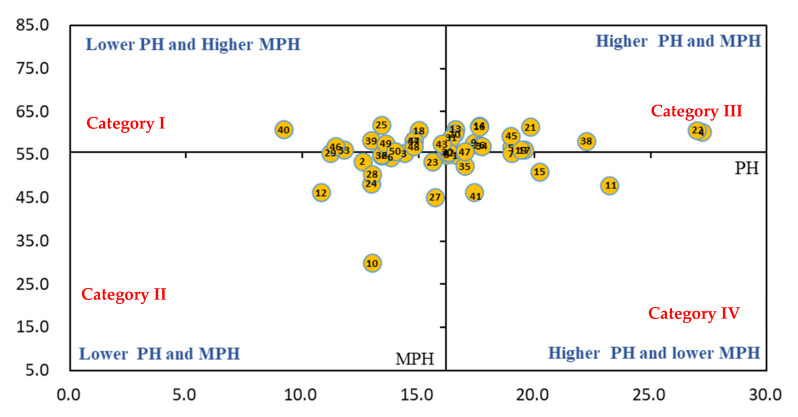
BCG matrix-based analysis of plant height (*n* = 50). Different numbers present alfalfa cultivars in this figure. PH: plant height before over-wintering in 2019; MPH: mean plant height of all six harvests.

As shown in Figure 6, there were 19 cultivars in Category I, namely, WL712HQ, WL440HQ, Eureka, and Vision, etc. Their plant heights before over-wintering were not high, but their mean plant heights in the second year were higher. There were 20 cultivars located in Category II, including WL903HQ, WL298HQ, Magnum7, and 4015, etc. These cultivars were not good, both in plant height before over-wintering and during the second year after over-wintering. There were seven cultivars in Category III, namely Magnum401, Alfalfa, Low TA, and Magnum7, etc. These cultivars had a higher plant height and a higher mean plant height in the second year. Furthermore, there were four cultivars in Category IV, namely Australia Queen, WL168HQ, SK4020, and Khan. They had higher plant heights before over-wintering, but low mean plant heights during the second year.

### 3.3. Canopy Area

Canopy area had significant variation among the six harvests when averaged across all cultivars (Figure 5). The canopy area of the first cutting was significantly higher (*p* < 0.05) than rest of the cuttings, followed by the fifth cutting. The order of canopy area was: first cutting > fifth cutting > fourth cutting > third cutting > sixth cutting > second cutting. Compared with the first cutting, canopy area of alfalfa in second, third, fourth, fifth, and sixth cuttings decreased by 63.8%, 36.9%, 34.8%, 32.2%, and 52.7%, respectively. Mean canopy area also varied significantly (*p* < 0.05) among cultivars, with the greatest mean canopy area measured for cultivars AC13, AC14, AC16, AC21, AC25, and AC40 (0.24 m^2^), while the smallest canopy area was measured for cultivar AC10 (0.09 m^2^) (Table 5).

**Figure 7:**
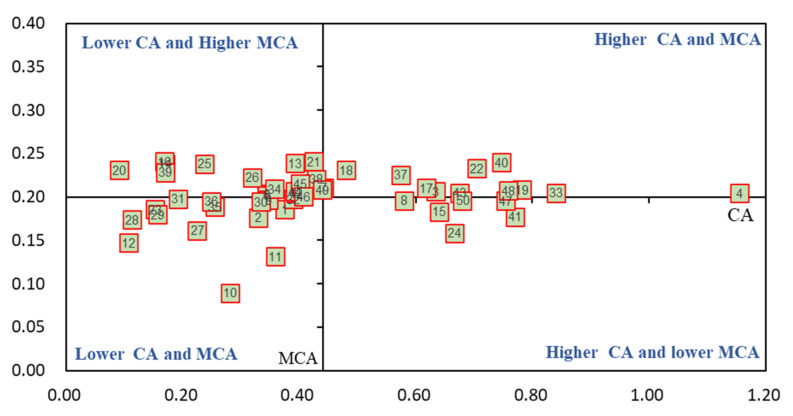
BCG matrix-based analysis of canopy area (*n* = 50). Different numbers present alfalfa cultivars in this figure. CA: canopy area before over-wintering in 2019; MCA: mean canopy area of all six harvests.

As shown in Figure 7, there were 23 cultivars in Category I, namely, Athena, Ladino, WL903HQ, and WL440, etc. The canopy area before over-wintering was not high, but their mean canopy area in the second year after over-wintering was higher. There were 10 cultivars located in Category II, including Guochan No. l, Magnum401, Australia Queen, and Low TA, etc. These cultivars were not good, both in canopy area before over-wintering and during the second year. There were 14 cultivars in Category III, namely WL366HQ, WL712HQ, Magnum7, and AC caribou, etc. These cultivars had a higher canopy area and a higher mean canopy area in the second year. Likewise, there are three cultivars in Category IV, namely WL168HQ, SK4030, and Khan. They had higher canopy area before over-wintering, but a low-mean canopy area during the second year.

### 3.4. Growth Rate

In the second cutting, the growth rate was significantly higher than the other cutting dates, and the sequence was as following: second cutting > fourth cutting > third cutting > fifth cutting > first cutting > sixth cutting (Figure 5). The growth rate of alfalfa in the first, third, fourth, fifth, and sixth decreased by 49.8%, 24.6%, 15.5%, 51.1%, and 50.8%, respectively, when compared with the second cutting. No significant differences in growth rates were observed among alfalfa cultivars during all cuttings (Table 5). The mean growth rate of alfalfa reached the maximum of 2.5 cm d^−1^ in AC43, AC44, and AC50 and the minimum of 1.3 cm d^−1^ in AC10.

**Figure 8:**
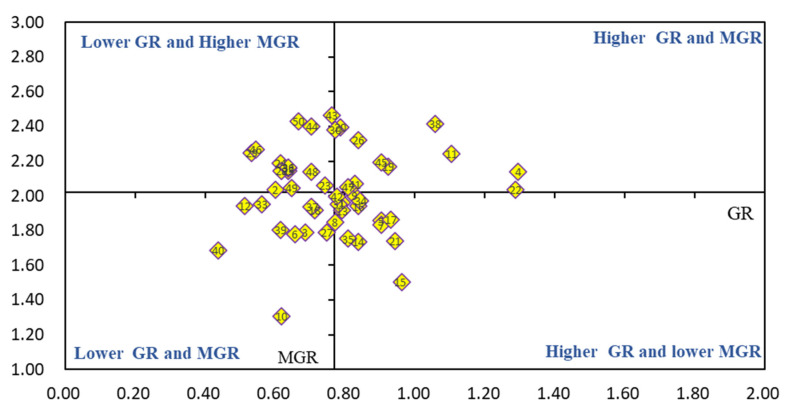
BCG matrix-based analysis of growth rate (*n* = 50). Different numbers present alfalfa cultivars in this figure. GR: growth rate before over-wintering in 2019; MGR: mean growth rate of all six harvests.

As shown in Figure 8, there were 15 cultivars in Category I, namely, Magnum7, Algonguin, Surprise, and WL363HQ, etc. The growth rate before over-wintering was not high, but their mean growth rate in the second year was higher. There were 17 cultivars located in Category II, including Inster, Alfalfa, Tango, and Low TA, etc. These cultivars were not good, both in growth rate before over-wintering and during the second year. There are nine cultivars in Category III, namely WL525HQ, Australia Queen, Seniti, and Weston, etc. These cultivars had a higher growth rate and a higher mean growth rate in the second year. Lastly, there were five cultivars in Category IV, namely FDA No.1, WL168HQ, WL366HQ, and WL903HQ. They had higher growth rates before over-wintering, but low-growth rates during the second year.

### 3.5. Comprehensive Evaluation

**Figure 9:**
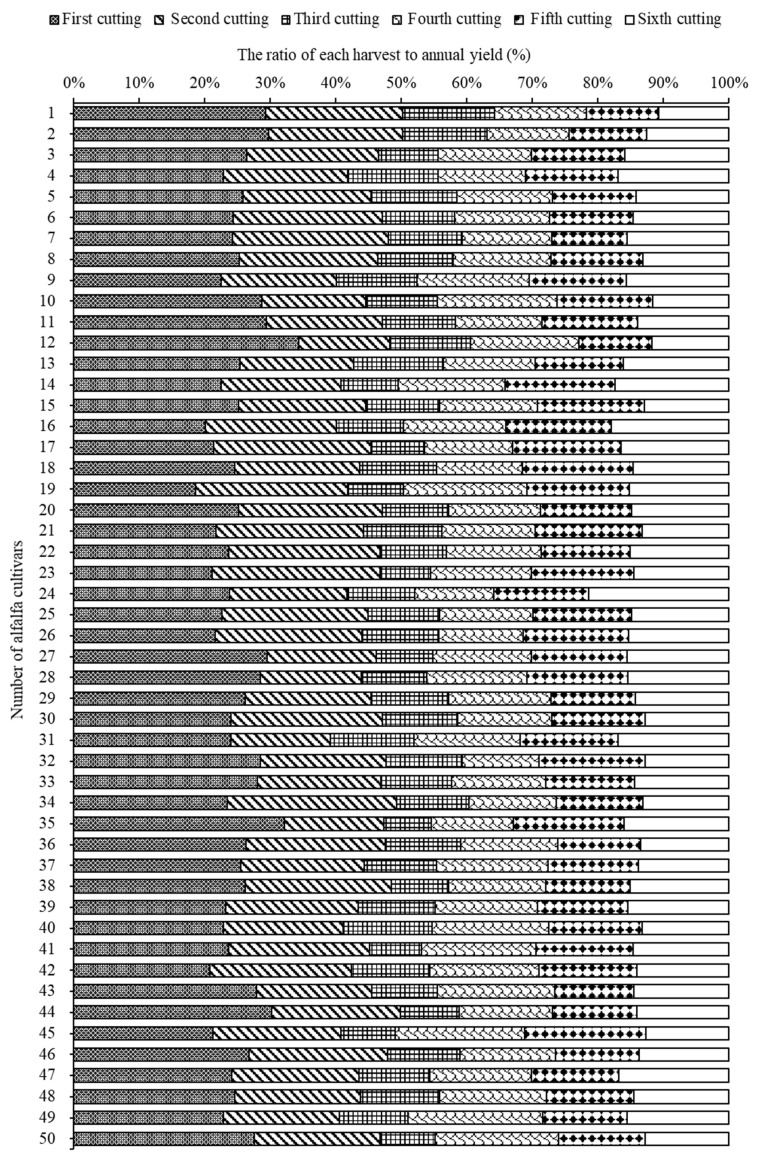
Percentage proportion of each harvest to total annual yield of different alfalfa cultivars.

**Figure 10:**
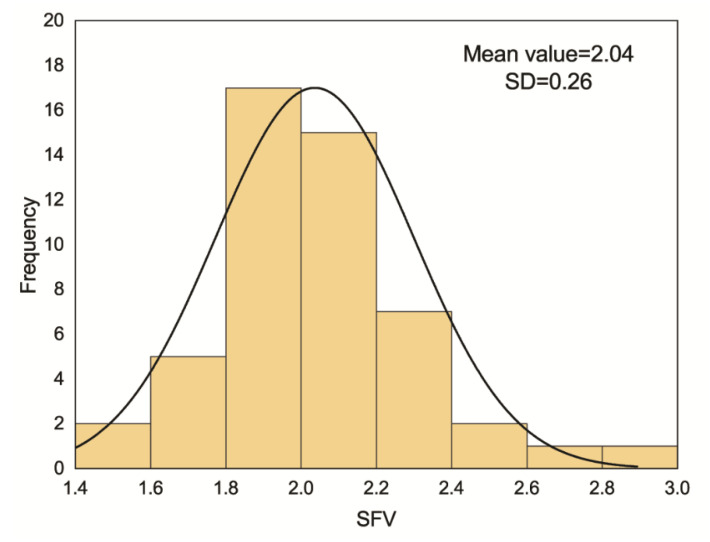
Distribution of subordinate function value of production adaptability among 50 cultivars or lines tested.

The subordinate function value (SFV) of all cultivars varied from 1.50 to 2.89; the maximum and minimum SFVs were observed in cultivar AC11 and AC8, respectively (Table 6). Among 50 alfalfa cultivars, five cultivars, AC8, AC25, AC26, AC48, and AC42, had better production adaptability. Meanwhile, cultivars AC11, AC10, AC43, AC4, AC47, and AC15 showed the lowest production adaptability. The distribution of SFV reflected the mean value and standard deviation of 2.04 and 0.26, respectively (Figure 9 and Figure 10).

**Figure 11:**
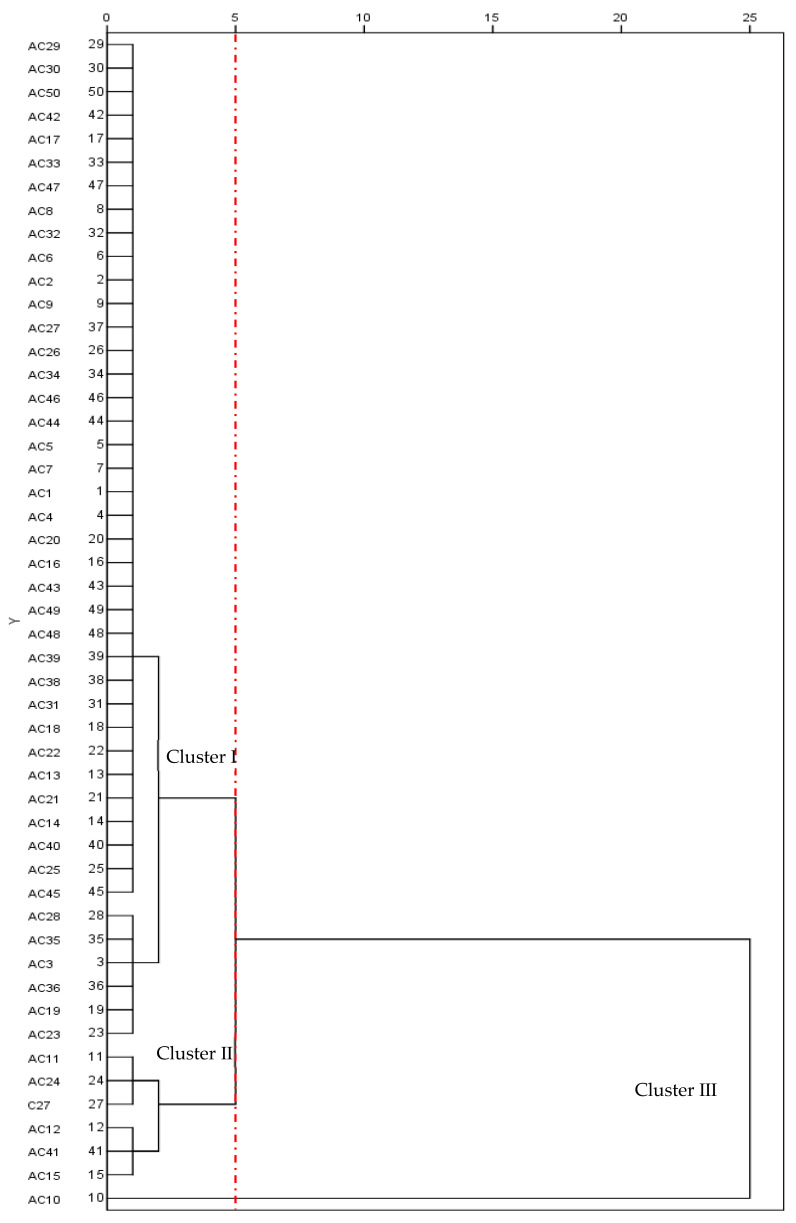
Cluster analysis for production adaptability of 50 alfalfa cultivars based on plant height, canopy area, growth rate, and mean annual yield.

Cluster analysis based on mean plant height, canopy area, growth rate, and total annual yield classified the tested 50 alfalfa cultivars into three groups (Figure 11). Cluster I was the group with highest plant height and canopy area, consisting of cultivars AC29, AC30, AC50, and AC42. The top five cultivars in terms of total annual yield were AC1, AC13, AC7, AC5, and AC21, and they were also ranked in Cluster I. Cluster II was the group with highest growth rate, consisting of cultivars AC11, AC24, AC27, AC12, AC41, and AC15. The cultivar AC10 had the lowest canopy area and growth rate, and thus it was placed into Cluster III.

## 4. Discussion

### 4.1. Forage Yield and Yield Components

Plant height, canopy area, growth rate, and forage yield in this study indicated that all tested cultivars had active growth during winter in this coastal region (Figure 2 and Figure 3). Alfalfa is usually harvested two to four times each year in the northern regions of China [30]. However, we found that a sixth harvest could be achieved in the coastal saline-alkali soil of North China and the highest forage yield was found in the first harvest. The reported annual alfalfa yield in our study (28.5 t ha^−1^) is higher than that reported in other studies conducted in the same region. Alfalfa annual forage yield varied from 10.6 to 14.4 t ha^−1^ in the Hebei area [31], and a low yield of 12 alfalfa cultivars with a maximum annual forage yield of 20.1 t ha^−1^ observed in Cangzhou City [32]. The forage yield of the Xunlu, WL656HQ, WL712HQ, WL363HQ, WL525HQ, and WL903HQ were 12.4, 12.6, 13.3, 12.4, 12.9, and 13.5 t ha^−1^ in study region, respectively [31]. These differences in forage yield may be due to variations in harvest frequencies and environmental conditions.

The mean plant height and canopy area of all cultivars at the first cutting were significantly taller (78.2 cm and 0.33 m^2^, respectively) than at all other cuttings. This could be due to the reason that cultivars took enough time (132 days) to achieve maximum growth before the second cutting, while all other cuttings got a shorter time (3–30 days) for growth and development compared with the first cutting. In the present study, canopy area and growth rate were strongly associated with forage yield, which implies that the level of canopy cover could represent the aerial biomass [33]. The above results indicate that the forage yield of alfalfa is positively correlated to canopy cover, and this could reduce temperature and transpiration [33,34,35].

### 4.2. Major Factors Influencing Production Adaptability of Alfalfa

Many factors affect the production adaptability of alfalfa including plant genotype, environmental conditions, field management practices, and stage of maturity [36,37]. The yield reduced by 42% in rainfed cropping compared to irrigation cropping [38]. Similar results were reported in another study [39]. Additionally, favourable air temperature and high precipitation during the alfalfa growing period contributed to the high alfalfa yield [12,40]. Drought and salt-stress-associated defoliation and diseases during summer resulted in plant death [41]. Air temperature and rainfall appeared to be major determinants on the growth of alfalfa cultivars in this study (Table 2), and the timing of consecutive harvests was dependent on the weather conditions during the alfalfa growing period [18]. This is not surprising because the major part of alfalfa growth in this study region occurs from May to September. This also emphasised the importance of rainfall on forage yield during the alfalfa growing period [21,42]. High temperature reduced forage yield, potentially because of reduced soil moisture due to high evaporation [21]. In this study, the precipitation amounts and mean temperatures were: 28.2, 5.6, 158.5, 324.5, and 73.7 mm, and 23.0, 26.9, 26.9, 27.0, and 22.9 °C from harvest one to six, respectively (Figure 1). The forage yield in the third cutting was the lowest, which might be related to the low precipitation. As coastal regions are usually characterised by high salinity and irregular rainfall, drought- and salt-tolerant cultivars should be selected for planting in order to ensure optimum forage yield. Alternating temperature, rainfall, and cultural practices may have a great impact on alfalfa growth, which ultimately results in the variation of the alfalfa yield. Further adoption of cultural practices aimed to improve forage yield may therefore be desired, including new cultivation models of inter-planting alfalfa with different crops for the decreasing of alfalfa canopy temperatures, and the further development and release of drought-tolerant cultivars for this study region.

Alfalfa forage yield is significantly influenced by the harvest intervals or cutting frequencies. Moreover, alfalfa growth is sensitive to the amount of growth days, and a short growing period would ultimately result in lower dry matter accumulation, which can be observed in the 23–30 days inter-harvest intervals for the second, third, fourth, fifth, and sixth cuttings in this study (Table 2). Forage yields with between 30- and 40-day harvest intervals were higher than the forage yield harvested with a 20-day interval [23]. Similar results regarding forage yield as being affected by harvest interval time were also reported [24,43]. In our study, although growth rate was the highest in the second harvest, with 23 days to reach blossom, forage yield was smaller than that in the first harvest. Similarly, the growth period was shorter from the third to sixth harvests than in other harvests which resulted in a smaller forage yield. Other researchers also noted a positive relationship between the forage yield and the regrowth period [44]. Therefore, in the coastal saline soil of Northern China, researchers should focus on the development of effective management practices for increasing alfalfa growth from the second to sixth harvests, which is of great importance to increasing the forage yield under such climate conditions.

**Figure 12:**
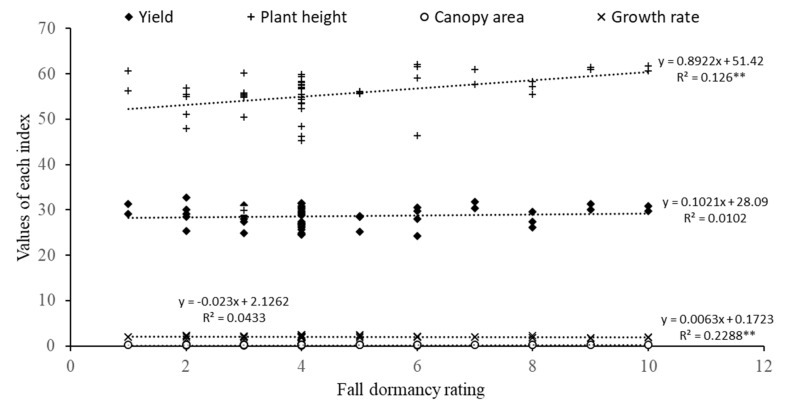
Regression analysis of fall dormancy rating vs. plant height, canopy area, growth rate, and mean annual yield during the six harvests. Coefficient of determination (*R^2^*) designated with “**” is significantly different (*p* < 0.05).

Additionally, regression analysis of the predicted fall dormancy rating vs. plant height and canopy area in this study resulted in high *R*^2^ values and positive slopes (plant height, *y* = 0.8922*x* + 51.42, *R*^2^ = 0.126 **; canopy area, *y* = 0.0063*x* + 0.1723, *R*^2^ = 0.2288 **) (Figure 12). However, the fall dormancy rating did not influence forage yield and growth rate, indicating that no effects of fall dormancy levels were observed on the forage yield in this study. In the present study, the three alfalfa cultivars with the highest annual yields ranging from 30.1 to 31.3 t ha^−1^ among fall dormancy ratings of 9–10 were selected from the evaluation of 50 cultivars. The main reason for this was the relatively high temperature in study region (Figure 1). The similar results were also obtained from another study [45]. The results of the fall dormancy ratings of the alfalfa cultivars were consistent with several previous studies [22,46,47].

## 5. Conclusions

In the present study, 50 alfalfa cultivars achieved very satisfactory total forage yields (ranging from 24.2 to 32.7 t ha^−1^) through their planting under non-irrigated rainfed conditions (748 mm precipitation and 14.4 °C temperature in the study area) in a coastal saline-alkali soil region. Six harvests of alfalfa could be achieved in this study region; however, the highest forage yield was found in the first harvest. We also found that there was great potential to improve the alfalfa forage yield from the second to sixth harvests because of the influence of air temperature and precipitation during the alfalfa growth period from May to September. Moreover, forage yield was negatively correlated with the growth rate. Importantly, cultivar Womu1 showed superior agronomic performance in terms of growth and forage yield. Future studies need to be conducted which focus on improving forage yields by adopting the rational cultivation model and inter-planting with different crops under optimal temperatures and water conditions in the saline soil regions.

## Figures and Tables

**Table 1:** Alfalfa cultivars and their origin.

No.	Cultivar’s Name	Code	Fall Dormaney Grade	Seed Source	Origin
1	Guochan No.1	AC1	2	Beijing Rytway Ecotechnology Co., Ltd.	China
2	Magnum401	AC2	4	Beijing Clover Seed Industry Co., Ltd.	America
3	Tango	AC3	8	Beijing Clover Seed Industry Co., Ltd.	Canada
4	WL525HQ	AC4	8.2	Beijing Rytway Ecotechnology Co., Ltd.	America
5	Magnum2	AC5	4	Beijing Clover Seed Industry Co., Ltd.	America
6	Inster	AC6	4	Beijing Clover Seed Industry Co., Ltd.	Canada
7	WL343	AC7	4	Beijing Rytway Ecotechnology Co., Ltd.	Canada
8	Womu No.1	AC8	3	Beijing Clover Seed Industry Co., Ltd.	China
9	Saidi No.7	AC9	7	Jiangxi Scarecrow Agriculture Park	Australia
10	Alfalfa	AC10	3	Jiangxi Scarecrow Agriculture Park	Australia
11	Australia Queen	AC11	2	Jiangxi Scarecrow Agriculture Park	Canada
12	Low TA	AC12	6	Jiangxi Scarecrow Agriculture Park	Australia
13	Athena	AC13	7	Jiangxi Scarecrow Agriculture Park	Australia
14	FDA No.10	AC14	10	Jiangxi Scarecrow Agriculture Park	Australia
15	WL168HQ	AC15	2	Beijing Rytway Ecotechnology Co., Ltd.	Canada
16	Southern Hemisphere	AC16	6	Jiangxi Scarecrow Agriculture Park	America
17	WL366HQ	AC17	5	Beijing Rytway Ecotechnology Co., Ltd.	America
18	WL712HQ	AC18	10.2	Beijing Rytway Ecotechnology Co., Ltd.	America
19	Seniti	AC19	5	Jiangxi Scarecrow Agriculture Park	France
20	Ladino	AC20	4	Beijing Rytway Ecotechnology Co., Ltd.	Australia
21	WL903HQ	AC21	9.5	Beijing Rytway Ecotechnology Co., Ltd.	America
22	WL298HQ	AC22	1	Beijing Rytway Ecotechnology Co., Ltd.	Canada
23	Magnum7-1	AC23	4	Beijing Clover Seed Industry Co., Ltd.	America
24	SK4030	AC24	4	Beijing Clover Seed Industry Co., Ltd.	Canada
25	WL440	AC25	6	Beijing Rytway Ecotechnology Co., Ltd.	America
26	Weston	AC26	8	Beijing Clover Seed Industry Co., Ltd.	America
27	Vienna	AC27	4	Jiangxi Scarecrow Agriculture Park	Canada
28	WL319HQ	AC28	3	Beijing Rytway Ecotechnology Co., Ltd.	Canada
29	Ameri graze 37CR	AC29	2	Beijing Clover Seed Industry Co., Ltd.	America
30	Magnum551	AC30	5	Beijing Clover Seed Industry Co., Ltd.	America
31	Magnum601	AC31	6	Beijing Clover Seed Industry Co., Ltd.	America
32	AC Caribou	AC32	3	Beijing Clover Seed Industry Co., Ltd.	Canada
33	Spade	AC33	1	Beijing Clover Seed Industry Co., Ltd.	Canada
34	WL358HQ	AC34	4	Beijing Rytway Ecotechnology Co., Ltd.	Canada
35	SK4020	AC35	4	Beijing Clover Seed Industry Co., Ltd.	Canada
36	Phabulous	AC36	4	Beijing Clover Seed Industry Co., Ltd.	Canada
37	Spade	AC37	4	Beijing Rytway Ecotechnology Co., Ltd.	Canada
38	Magnum7-2	AC38	4	Beijing Clover Seed Industry Co., Ltd.	America
39	Eureka	AC39	8	Jiangxi Scarecrow Agriculture Park	Australia
40	WL656HQ	AC40	9.3	Beijing Rytway Ecotechnology Co., Ltd.	America
41	Khan	AC41	4	Beijing Rytway Ecotechnology Co., Ltd.	Canada
42	WL354HQ	AC42	3	Beijing Rytway Ecotechnology Co., Ltd.	Canada
43	SK3010	AC43	2.5	Beijing Clover Seed Industry Co., Ltd.	Canada
44	Magnum7-3	AC44	4	Beijing Clover Seed Industry Co., Ltd.	America
45	4015	AC45	4	Beijing Clover Seed Industry Co., Ltd.	America
46	Algonguin	AC46	2	Beijing Rytway Ecotechnology Co., Ltd.	Canada
47	Pioneer	AC47	3	Beijing Rytway Ecotechnology Co., Ltd.	Canada
48	Surprise	AC48	4	Beijing Rytway Ecotechnology Co., Ltd.	Canada
49	Vision	AC49	4	Beijing Clover Seed Industry Co., Ltd.	Canada
50	WL363HQ	AC50	5	Beijing Rytway Ecotechnology Co., Ltd.	Canada

**Table 2:** The dates of sowing time, seedling emergence, early flowering, and first cutting of 50 alfalfa cultivars during 2019–2020.

Cultivar’s Name	Sowing Time	Seedling Emergence	Early Flowering	First Cutting
Guochan No.1	15 August 2019	21 August 2019	14 May 2020	16 May 2020
Magnum401	15 August 2019	21 August 2019	14 May 2020	16 May 2020
Tango	15 August 2019	21 August 2019	10 May 2020	12 May 2020
WL525HQ	15 August 2019	20 August 2019	11 May 2020	12 May 2020
Magnum2	15 August 2019	21 August 2019	14 May 2020	15 May 2020
Inster	15 August 2019	21 August 2019	14 May 2020	16 May 2020
WL343	15 August 2019	24 August 2019	14 May 2020	16 May 2020
Womu No.1	15 August 2019	21 August 2019	14 May 2020	16 May 2020
Saidi No.7	15 August 2019	21 August 2019	16 May 2020	17 May 2020
Alfalfa	15 August 2019	21 August 2019	17 May 2020	18 May 2020
Australia Queen	15 August 2019	21 August 2019	14 May 2020	16 May 2020
Low TA	15 August 2019	21 August 2019	10 May 2020	12 May 2020
Athena	15 August 2019	21 August 2019	10 May 2020	12 May 2020
FDA No.10	16 August 2019	21 August 2019	10 May 2020	12 May 2020
WL168HQ	16 August 2019	24 August 2019	14 May 2020	16 May 2020
Southern Hemisphere	16 August 2019	22 August 2019	11 May 2020	12 May 2020
WL366HQ	16 August 2019	24 August 2019	11 May 2020	12 May 2020
WL712HQ	16 August 2019	24 August 2019	14 May 2020	16 May 2020
Seniti	16 August 2019	21 August 2019	14 May 2020	16 May 2020
Ladino	16 August 2019	26 August 2019	11 May 2020	12 May 2020
WL903HQ	16 August 2019	23 August 2019	10 May 2020	12 May 2020
WL298HQ	16 August 2019	23 August 2019	10 May 2020	12 May 2020
Magnum7-1	16 August 2019	23 August 2019	15 May 2020	16 May 2020
SK4030	16 August 2019	26 August 2019	15 May 2020	17 May 2020
WL440	16 August 2019	23 August 2019	15 May 2020	16 May 2020
Weston	16 August 2019	23 August 2019	14 May 2020	16 May 2020
Vienna	16 August 2019	21 August 2019	14 May 2020	16 May 2020
WL319HQ	16 August 2019	22 August 2019	14 May 2020	16 May 2020
Ameri graze 37CR	16 August 2019	22 August 2019	15 May 2020	16 May 2020
Magnum551	16 August 2019	26 August 2019	15 May 2020	16 May 2020
Magnum601	16 August 2019	26 August 2019	10 May 2020	12 May 2020
AC Caribou	16 August 2019	22 August 2019	10 May 2020	12 May 2020
Spade	16 August 2019	27 August 2019	14 May 2020	16 May 2020
WL358HQ	16 August 2019	26 August 2019	11 May 2020	12 May 2020
SK4020	16 August 2019	27 August 2019	14 May 2020	16 May 2020
Phabulous	16 August 2019	23 August 2019	14 May 2020	16 May 2020
Spade	16 August 2019	26 August 2019	14 May 2020	16 May 2020
Magnum7-2	16 August 2019	25 August 2019	14 May 2020	16 May 2020
Eureka	16 August 2019	24 August 2019	11 May 2020	12 May 2020
WL656HQ	16 August 2019	26 August 2019	14 May 2020	12 May 2020
Khan	17 August 2019	25 August 2019	14 May 2020	16 May 2020
WL354HQ	17 August 2019	27 August 2019	14 May 2020	16 May 2020
SK3010	16 August 2019	25 August 2019	14 May 2020	16 May 2020
Magnum7-3	16 August 2019	26 August 2019	14 May 2020	16 May 2020
4015	17 August 2019	25 August 2019	14 May 2020	16 May 2020
Algonguin	17 August 2019	25 August 2019	14 May 2020	16 May 2020
Pioneer	17 August 2019	24 August 2019	15 May 2020	16 May 2020
Surprise	17 August 2019	25 August 2019	15 May 2020	16 May 2020
Vision	17 August 2019	25 August 2019	14 May 2020	16 May 2020
WL363HQ	17 August 2019	27 August 2019	15 May 2020	16 May 2020

**Table 3:** Total annual forage yield of different alfalfa cultivars and their ranking.

Cultivar Name	Forage Yield (t ha^−1^)	Rank	Cultivar Name	Forage Yield (t ha^−1^)	Rank
Guochan No.1	32.7 ± 1.7 ^a^	1	Weston	29.5 ± 3.1 ^abcdefg^	21
Magnum401	29.8 ± 2.5 ^abcdef^	17	Vienna	24.5 ± 3.2 ^fg^	48
Tango	26.1 ± 2.6 ^bcdefg^	42	WL319HQ	24.9 ± 2.1 ^efg^	46
WL525HQ	28.0 ± 2.6 ^abcdef^	32	Ameri graze 37CR	28.4 ± 2.3 ^abcdefg^	30
Magnum2	31.4 ± 2.5 ^ab^	4	Magnum551	28.5 ± 3.7 ^abcdefg^	29
Inster	30.5 ± 4.1 ^abcdef^	11	Magnum601	24.2 ± 6.3 ^g^	50
WL343	31.4 ± 3.6 ^ab^	3	AC Caribou	30.0 ± 3.1 ^abcdefg^	16
Womu No.1	29.8 ± 2.1 ^abcdef^	18	Spade	29.1 ± 2.1 ^abcdefg^	25
Saidi No.7	30.4 ± 1.4 ^abcdef^	12	WL358HQ	29.4 ± 2.6 ^abcdefg^	22
Alfalfa	31.0 ± 3.2 ^abcd^	7	SK4020	24.9 ± 3.9 ^efg^	47
Australia Queen	25.3 ± 1.8 ^cdefg^	44	Phabulous	26.2 ± 3.6 ^bcdefg^	41
Low TA	29.7 ± 3.1 ^abcdefg^	19	Spade	30.2 ± 3.3 ^abcdefg^	13
Athena	31.7 ± 3.6 ^ab^	2	Magnum7-2	25.7 ± 3.0 ^bcdefg^	43
FDA No.10	29.7 ± 4.1 ^abcdefg^	20	Eureka	27.4 ± 3.2 ^abcdefg^	34
WL168HQ	30.1 ± 2.3 ^abcdefg^	15	WL656HQ	30.1 ± 3.1 ^abcdefg^	14
Southern Hemisphere	28.0 ± 2.2 ^abcdefg^	33	Khan	29.1 ± 1.9 ^abcdefg^	24
WL366HQ	28.4 ± 4.4 ^abcdefg^	31	WL354HQ	28.7 ± 2.4 ^abcdefg^	27
WL712HQ	30.9 ± 2.9 ^abcde^	8	SK3010	27.0 ± 1.0 ^abcdefg^	37
Seniti	25.2 ± 1.6 ^defg^	45	Magnum7-3	28.8 ± 2.9 ^abcdefg^	26
Ladino	26.8 ± 2.5 ^abcdefg^	39	4015	30.9 ± 2.9 ^abcde^	9
WL903HQ	31.3 ± 2.7 ^abc^	5	Algonguin	29.1 ± 2.8 ^abcdefg^	23
WL298HQ	31.3 ± 3.8 ^abc^	6	Pioneer	27.4 ± 1.9 ^abcdefg^	35
Magnum7-1	26.9 ± 2.6 ^abcdefg^	38	Surprise	27.4 ± 3.0 ^abcdefg^	36
SK4030	24.5 ± 2.9 ^fg^	49	Vision	26.4 ± 2.4 ^bcdefg^	40
WL440	30.5 ± 2.6 ^abcdef^	10	WL363HQ	28.7 ± 2.2 ^abcdefg^	28

Note: Different lowercase letters represent significantly different means among alfalfa cultivars at 0.05.

**Table 4:** Pearson correlation coefficients among forage yield and agronomic traits of alfalfa.

Agronomic Traits	Total Annual Yield	Plant Height	Canopy Area
Plant height	0.164		
Canopy area	0.237	0.926 **	
Growth rate	−0.300 *	0.312 *	0.140

Note: *, **: significant at 0.05 and 0.01 probability levels, respectively.

**Table 5:** Growth characteristics (i.e., plant height, canopy area, and growth rate) of different alfalfa cultivars under field conditions.

No.	Plant Height (cm)	Canopy Area (m^2^)	Growth Rate (cm d^−1^)	No.	Plant Height (cm)	Canopy Area (m^2^)	Growth Rate (cm d^−1^)
1	55.0 ± 12.9 ^abcd^	0.19 ± 0.10 ^ab^	2.0 ± 0.6 ^a^	26	57.2 ± 6.6 ^abcd^	0.22 ± 0.05 ^ab^	2.3 ± 1.0 ^a^
2	53.5 ± 10.0 ^abcd^	0.18 ± 0.09 ^abc^	2.0 ± 0.7 ^a^	27	45.2 ± 16.1 ^d^	0.16 ± 0.10 ^abc^	1.8 ± 1.1 ^a^
3	55.4 ± 9.6 ^abcd^	0.21 ± 0.06 ^ab^	1.8 ± 0.7 ^a^	28	50.5 ± 10.5 ^abcd^	0.17 ± 0.08 ^abc^	2.1 ± 0.7 ^a^
4	60.2 ± 8.2 ^abcd^	0.20 ± 0.09 ^ab^	2.1 ± 0.9 ^a^	29	55.5 ± 9.7 ^abcd^	0.18 ± 0.06 ^abc^	2.2 ± 0.8 ^a^
5	56.7 ± 10.6 ^abcd^	0.20 ± 0.07 ^ab^	1.9 ± 0.6 ^a^	30	55.6 ± 9.0 ^abcd^	0.19 ± 0.06 ^ab^	2.4 ± 1.1 ^a^
6	54.4 ± 9.0 ^abcd^	0.20 ± 0.05 ^ab^	1.8 ± 0.4 ^a^	31	59.0 ± 8.2 ^abcd^	0.20 ± 0.05 ^ab^	2.0 ± 0.7 ^a^
7	55.4 ± 9.8 ^abcd^	0.21 ± 0.05 ^ab^	1.8 ± 0.5 ^a^	32	54.9 ± 8.8 ^abcd^	0.20 ± 0.07 ^ab^	2.2 ± 1.3 ^a^
8	55.1 ± 10.4 ^abcd^	0.20 ± 0.07 ^ab^	1.8 ± 0.6 ^a^	33	56.2 ± 9.8 ^abcd^	0.20 ± 0.06 ^ab^	2.0 ± 0.7 ^a^
9	57.7 ± 10.1 ^abcd^	0.20 ± 0.09 ^ab^	2.0 ± 0.6 ^a^	34	57.0 ± 7.3 ^abcd^	0.21 ± 0.05 ^ab^	2.0 ± 1.1 ^a^
10	29.9 ± 9.6 ^e^	0.09 ± 0.10 ^c^	1.3 ± 1.0 ^a^	35	52.4 ± 9.7 ^abcd^	0.19 ± 0.05 ^ab^	1.8 ± 0.5 ^a^
11	48.0 ± 9.5 ^abcd^	0.13 ± 0.07^ bc^	2.2 ± 1.7 ^a^	36	54.9 ± 7.3 ^abcd^	0.20 ± 0.05 ^ab^	2.2 ± 0.9 ^a^
12	46.3 ± 15.2 ^bcd^	0.15 ± 0.10 ^abc^	1.9 ± 1.1 ^a^	37	58.3 ± 9.7 ^abcd^	0.23 ± 0.05 ^a^	1.9 ± 0.8 ^a^
13	60.9 ± 7.3 ^abc^	0.24 ± 0.05 a	1.9 ± 1.0 ^a^	38	58.2 ± 7.3 ^abcd^	0.22 ± 0.05 ^ab^	2.4 ± 1.5 ^a^
14	61.7 ± 11.5 ^a^	0.24 ± 0.06 a	1.7 ± 0.5 ^a^	39	58.3 ± 8.6 ^abcd^	0.23 ± 0.05 ^a^	1.9 ± 1.0 ^a^
15	51.1 ± 16.6 ^abcd^	0.18 ± 0.09 ab	1.5 ± 0.5 ^a^	40	60.9 ± 7.1 ^abc^	0.24 ± 0.04 ^a^	1.6 ± 0.9 ^a^
16	61.5 ± 11.6 ^ab^	0.24 ± 0.06 a	1.9 ± 0.4 ^a^	41	46.2 ± 19.4 ^cd^	0.18 ± 0.08 ^abc^	2.1 ± 0.8 ^a^
17	56.1 ± 9.8 ^abcd^	0.21 ± 0.06 ab	1.9 ± 0.6 ^a^	42	55.5 ± 7.7 ^abcd^	0.20 ± 0.05 ^ab^	2.1 ± 0.7 ^a^
18	60.6 ± 8.3 ^abc^	0.23 ± 0.06 a	1.9 ± 0.9 ^a^	43	57.5 ± 8.7 ^abcd^	0.20 ± 0.06 ^ab^	2.5 ± 0.8 ^a^
19	56.1 ± 8.6 ^abcd^	0.21 ± 0.05 ab	2.2 ± 0.7 ^a^	44	58.3 ± 6.8 ^abcd^	0.21 ± 0.06 ^ab^	2.5 ± 0.9 ^a^
20	59.9 ± 7.1 ^abcd^	0.23 ± 0.05 a	2.4 ± 1.1 ^a^	45	59.4 ± 7.3 ^abcd^	0.22 ± 0.06 ^ab^	2.3 ± 0.8 ^a^
21	61.4 ± 8.5 ^abc^	0.24 ± 0.05 ^a^	1.7 ± 0.8 ^a^	46	56.9 ± 10.8 ^abcd^	0.20 ± 0.07 ^ab^	2.3 ± 0.4 ^a^
22	60.7 ± 7.0 ^abc^	0.23 ± 0.05 ^a^	2.0 ± 0.8 ^a^	47	55.7 ± 10.5 ^abcd^	0.20 ± 0.06 ^ab^	2.1 ± 0.5 ^a^
23	53.4 ± 9.5 ^abcd^	0.19 ± 0.06 ^ab^	2.1 ± 1.1 ^a^	48	56.9 ± 9.0 ^abcd^	0.21 ± 0.05 ^ab^	2.1 ± 0.4 ^a^
24	48.4 ± 11.0 ^abcd^	0.16 ± 0.07 ^abc^	2.2 ± 1.0 ^a^	49	57.6 ± 10.3 ^abcd^	0.21 ± 0.07 ^ab^	2.2 ± 0.7 ^a^
25	62.0 ± 9.6 ^a^	0.24 ± 0.06 ^a^	2.1 ± 0.8 ^a^	50	55.8 ± 9.8 ^abcd^	0.20 ± 0.06 ^ab^	2.5 ± 0.9 ^a^

Note: Different lowercase letters represent significantly different means among alfalfa cultivars at 0.05.

**Table 6:** Subordinate function values of different alfalfa cultivars and their rankings.

Code	Plant Height	Canopy Area	Growth Rate	Annual Yield	Evaluation	Rank
AC1	0.42	0.49	0.52	0.50	1.93	34
AC2	0.52	0.50	0.43	0.39	1.84	40
AC3	0.55	0.59	0.31	0.38	1.83	41
AC4	0.52	0.60	0.33	0.26	1.71	47
AC5	0.53	0.41	0.49	0.40	1.83	42
AC6	0.56	0.52	0.43	0.47	1.98	27
AC7	0.53	0.54	0.55	0.44	2.06	20
AC8	0.51	0.49	0.35	1.54	2.89	1
AC9	0.53	0.57	0.56	0.37	2.03	22
AC10	0.31	0.28	0.44	0.49	1.52	49
AC11	0.40	0.41	0.34	0.35	1.50	50
AC12	0.36	0.37	0.38	0.75	1.86	39
AC13	0.59	0.62	0.35	0.51	2.07	19
AC14	0.64	0.61	0.42	0.31	1.98	28
AC15	0.54	0.51	0.30	0.38	1.73	45
AC16	0.66	0.61	0.48	0.37	2.12	14
AC17	0.56	0.55	0.37	0.41	1.89	37
AC18	0.49	0.57	0.38	0.49	1.93	35
AC19	0.68	0.65	0.43	0.36	2.12	15
AC20	0.82	0.75	0.38	0.38	2.33	7
AC21	0.55	0.56	0.47	0.36	1.94	31
AC22	0.68	0.64	0.32	0.45	2.09	17
AC23	0.31	0.38	0.45	0.91	2.05	21
AC24	0.65	0.43	0.55	0.45	2.08	18
AC25	0.61	0.61	0.51	0.92	2.65	2
AC26	0.70	0.70	0.32	0.72	2.44	3
AC27	0.43	0.34	0.34	0.84	1.95	30
AC28	0.63	0.51	0.26	0.48	1.88	38
AC29	0.55	0.44	0.34	0.48	1.81	43
AC30	0.59	0.56	0.35	0.53	2.03	23
AC31	0.63	0.46	0.38	0.55	2.02	25
AC32	0.50	0.48	0.39	0.52	1.89	36
AC33	0.50	0.47	0.47	0.50	1.94	32
AC34	0.69	0.44	0.59	0.47	2.19	13
AC35	0.67	0.57	0.47	0.57	2.28	9
AC36	0.61	0.51	0.40	0.72	2.24	10
AC37	0.54	0.56	0.38	0.55	2.03	24
AC38	0.46	0.54	0.29	0.64	1.93	33
AC39	0.60	0.55	0.62	0.42	2.19	12
AC40	0.53	0.55	0.43	0.59	2.10	16
AC41	0.62	0.48	0.55	0.57	2.22	11
AC42	0.71	0.65	0.47	0.55	2.38	5
AC43	0.48	0.41	0.30	0.51	1.70	48
AC44	0.57	0.52	0.34	0.36	1.79	44
AC45	0.79	0.64	0.50	0.44	2.37	6
AC46	0.47	0.41	0.64	0.49	2.01	26
AC47	0.56	0.48	0.38	0.30	1.72	46
AC48	0.68	0.52	0.67	0.56	2.43	4
AC49	0.68	0.60	0.44	0.60	2.32	8
AC50	0.69	0.55	0.39	0.34	1.97	29

## Data Availability

The authors confirm that the data supporting the findings of this study are available within the article.
